# Impairing the maintenance of germinative cells in *Echinococcus multilocularis* by targeting Aurora kinase

**DOI:** 10.1371/journal.pntd.0007425

**Published:** 2019-05-16

**Authors:** Zhe Cheng, Fan Liu, Huimin Tian, Zhijian Xu, Xiaoli Chai, Damin Luo, Yanhai Wang

**Affiliations:** 1 State Key Laboratory of Cellular Stress Biology, School of Life Sciences, Xiamen University, Xiamen, Fujian, China; 2 Parasitology Research Laboratory, School of Life Sciences, Xiamen University, Xiamen, Fujian, China; 3 Medical College, Xiamen University, Xiamen, Fujian, China; University of Würzburg, GERMANY

## Abstract

**Background:**

The tumor-like growth of the metacestode larvae of the tapeworm *E*. *multilocularis* causes human alveolar echinococcosis, a severe disease mainly affecting the liver. The germinative cells, a population of adult stem cells, are crucial for the larval growth and development of the parasite within the hosts. Maintenance of the germinative cell pools relies on their abilities of extensive proliferation and self-renewal, which requires accurate control of the cell division cycle. Targeting regulators of the cell division progression may impair germinative cell populations, leading to impeded parasite growth.

**Methodology/Principal findings:**

In this study, we describe the characterization of EmAURKA and EmAURKB, which display significant similarity to the members of Aurora kinases that are essential mitotic kinases and play key roles in cell division. Our data suggest that EmAURKA and EmAURKB are actively expressed in the germinative cells of *E*. *multilocularis*. Treatment with low concentrations of MLN8237, a dual inhibitor of Aurora A and B, resulted in chromosomal defects in the germinative cells during mitosis, while higher concentrations of MLN8237 caused a failure in cytokinesis of the germinative cells, leading to multinucleated cells. Inhibition of the activities of Aurora kinases eventually resulted in depletion of the germinative cell populations in *E*. *multilocularis*, which in turn caused larval growth inhibition of the parasite.

**Conclusions/Significance:**

Our data demonstrate the vital roles of Aurora kinases in the regulation of mitotic progression and maintenance of the germinative cells in *E*. *multilocularis*, and suggest Aurora kinases as promising druggable targets for the development of novel chemotherapeutics against human alveolar echinococcosis.

## Author summary

Alveolar echinococcosis (AE), caused by infection with the metacestode larvae of the tapeworm *E*. *multilocularis*, is a lethal disease in humans. A population of adult stem cells, called germinative cells, drive the cancer-like growth of the parasite within their host and are considered responsible for disease recurrence after therapy termination. Nevertheless, benzimidazoles, the current drugs of choice against AE, show limited effects on killing these cells. Here, we describe EmAURKA and EmAURKB, two Aurora kinase members that play essential roles in regulating *E*. *multilocularis* germinative cell mitosis, as promising drug targets for eliminating the population of germinative cells. We show that targeting *E*. *multilocularis* Aurora kinases by small molecular inhibitor MLN8237 causes severe mitotic defects and eventually impairs the viability of germinative cells, leading to larval growth inhibition of the parasite *in vitro*. Our study suggests that targeting mitosis by MLN8237 or related compounds offers possibilities for germinative cell killing and we hope this will help in exploring novel therapeutic strategies against the disease.

## Introduction

Alveolar echinococcosis (AE), caused by the larval stage of the cestode *Echinococcus multilocularis*, is considered as the most lethal helminthiasis. Humans become infected by accidentally ingesting infectious eggs, which contain oncospheres and then develop into cyst-like metacestode vesicles mainly in the liver. The metacestode larvae grow multivesicularly and infiltratively like tumors in host tissue, eventually leading to organ failure. The protoscoleces are generated in the metacestode vesicles through asexual multiplication, and then either mature into adult tapeworms if ingested by the definitive host (canids) or develop into metacestode vesicles when distributed in the intermediate host [1].

The ideal option for human AE treatment to date is surgery, which is always accompanied by chemotherapy. In cases where surgery is not possible, chemotherapy remains the only option [2]. Current anti-AE chemotherapy mainly relies on the benzimidazole carbamate derivatives albendazole and mebendazole. However, these drugs are only parasitostatic rather than parasiticidal, and the treatment usually needs years or even the life-long uptake of drugs. In addition, severe adverse side effects and the intolerance of benzimidazoles for a number of patients also limit their use for AE treatment. Therefore, novel chemotherapeutic options against AE have to be pursued [3–4].

It has been demonstrated that *E*. *multilocularis* possesses a population of pluripotent stem cells, called germinative cells. These cells are the only type of cells capable of proliferation and they give rise to all differentiated cells in the parasite. Hence, germinative cells are decisive for the tumor-like, infiltrative growth of *E*. *multilocularis* larvae within host organs, and should also be responsible for parasite recurrence upon discontinuation of chemotherapy [5–6]. Due to its fundamental roles in the asexual multiplication of *E*. *multilocularis* metacestode, the population of germinative cells has emerged as a crucial target to be considered for the development of chemotherapeutics against AE [6].

Aurora kinases (AURK), a family of serine/threonine kinases, play pivotal roles in the control of cell division via regulating mitosis especially chromosomal segregation and cytokinesis [7–10]. They have been described in various organisms, and their structure and function are well conserved through evolution. Yeast just has one Aurora kinase, while metazoans generally have two, named Aurora A and Aurora B. A third family member, Aurora C, is unique for mammals [11–13]. Although the N-terminus of AURK is variable among organisms, the C-terminal catalytic domain that contains the activation loop (T-loop) and the degradation box (D-box) is highly conserved within the family. Despite significant sequence homology, the localization and function of AURK members are largely distinct from one another. In mitotic cells, Aurora A localizes to the centrosomes and spindle microtubules, and functions in centrosome maturation, mitotic entry, and spindle assembly. Aurora B localizes to the inner centromere and spindle midzone, and is mainly involved in spindle assembly checkpoint, kinetochore attachment, and cytokinesis. Aurora C is expressed in testis, where it exhibits tissue-specific functions [7, 9, 11–13].

Dysfunction of Aurora kinases causes mitotic errors, which leads to genetic instability and chromosomal aneuploidy. Increasing evidence has shown that Aurora A and B are deregulated and/or overexpressed in many kinds of human tumors, and that inhibition of Aurora kinase results in cancer cell mitotic arrest and cell death [7, 8]. Aurora kinases have therefor emerged as attractive targets for cancer therapy, and a number of Aurora kinase inhibitors are developed and approved for various stages of clinical testing [10, 14, 15]. Strikingly, Aurora kinases have been identified in protozoan parasites (e.g. *Trypanosoma brucei*, *Leishmania major* and *Plasmodium falciparum*) [16–19], and human Aurora inhibitors have been shown to effectively inhibit the proliferation of these parasites, leading to Aurora kinase as the promising anti-protozoan drug targets [20–23].

In this study we identified two members of Aurora kinase in *E*. *multilocularis*, EmAURKA and EmAURKB, and show that they are actively expressed in the germinative cells. Targeting *E*. *multilocularis* Aurora kinases by MLN8237 causes severe mitotic defects and impairs the maintenance of germinative cell populations that leads to larval growth inhibition of the parasite, suggesting Aurora kinases as druggable targets for the development of chemotherapeutics against AE.

## Methods

### Ethics statement

All animal experiments were conducted in strict accordance with China regulations on the protection of experimental animals (Regulations for the Administration of Affairs Concerning Experimental Animals, version from July-18-2013) and specifically approved by the Institutional Animal Care and Use Committee of Xiamen University (Permit Number: 2013–0053).

### Identification and cloning of Aurora kinase genes of *E. multilocularis*

Published sequences of the Aurora kinase members of human, *Xenopus*, zebrafish, *Drosophila* and *C*. *elegans* were used as queries to blast the *E*. *multilocularis* genome database [24] available at http://www.sanger.ac.uk/resources/downloads/helminths/echinococcusmultilocularis. Only two gene loci (EmuJ_001059700 (EmAurka) and EmuJ_000891900 (EmAurkb)) encoding the members of Aurora kinase family were identified and their full coding sequences were then amplified from the cDNA preparations as described before [25]. 5’-rapid amplification of cDNA ends (RACE) was performed using the SMART RACE cDNA Amplification Kit (Clontech) according to the manufacturer’s instructions. Kinase domain was determined using the online software SMART (http://smart.embl-heidelberg.de/). Primers for amplification of the full coding sequences of *EmAurka* and *EmAurkb* were used as follows: EmAurka-F (5’-ATG CGT ATT ATG GAC GAC TCT GCT TTT CCC GAT-3’), EmAurka-R (5’-TTA AGT TCT TGT CGA GCT GGG GGT GGA GGC-3’), EmAurkb-F (5’-ATG AGT TCC TTG ATC GAA TAC GGT ACC CCT TC-3’) and EmAurkb-R (5’- TCA TGG TGG TGG CTT GCC CCT TTC GG-3’).

### Parasite in vitro cultivation and drug treatment

Parasite was maintained by *in vivo* propagation of the parasite material in mice (supplied by Xiamen University Laboratory Animals Center, XMULAC). Mature and developing protoscoleces were collected from parasite material, manually picked under the microscope, and then immediately used for RNA isolation or EdU labeling. *In vitro* cultivation of metacestode vesicles was performed using host cell conditioned medium as previously described [26]. The growth of metacestode vesicles and the process of vesicle formation from protoscoleces were examined after 21 days and 14 days of culture, respectively as described by Cheng et al. [27].

Aurora inhibitors (MLN8237, MLN8054, MK-5108 and AZD1152-HQPA), nocodazole and hydroxyurea were supplied by Selleck Chemicals. Drugs were added into the culture medium at a final concentration as indicated. All of the drug experiments were carried out under axenic culture conditions as described before [26]. For longer periods of treatment, experiments were performed with exchange of the medium containing the same ingredients every three days.

### mRNA expression analysis of *EmAurka* and *EmAurkb*

Total RNA was extracted from different larval stages of the parasite or the *in vitro*-cultivated metacestode vesicles treated with 40 mM of hydroxyurea or not. RNA was purified after DNase treatment and then reverse transcribed into cDNA. cDNAs were processed for real-time quantitative PCR (qPCR) analysis using the primers: 59700-qF (5’- TAA AGC GAG TGT TGG AAA -3’) and 59700-qR (5’- GCA GGC TGA CAT GAA AGT-3’) for *EmAurka*; 891900-qF (5’- GTC GGA GTT TTG TGC T-3’) and 891900-qR (5’- GAT CTT CGA AAT CAG GTC-3’) for *EmAurkb*; ELP-DW (5’-CAG GAT CTC TTC GAT CAA GTG-3’) and ELP-UP (5’-CCT GTG TTG CCA AGT ATG GTC-3’) for the constitutively expressed gene *elp* as the internal control [28].

### *In vitro* kinase assay

The inactive mutants EmAURKA^T208A^ and EmAURKB^T184A^ were generated by substituting alanine for threonine in the conserved RxT motif within the Aurora kinase domain. pcDNA3.3-HA plasmids (gifts from Prof. Han Jiahuai, Xiamen University, China) containing the reading frame of *EmAurka* or *EmAurkb* were used as the templates, and the mutants were obtained by mutagenesis PCR as previously described [29] using the following primers: T208A-F (5’-CCA GGT TCT AGG CGT GCT GCT GTT TGG-3’) and T208A-R (5’- CAC GCC TAG AAC CTG GGC TGT GGA CCG CAC-3’) for EmAURKA^T208A^; T184A-F (5’-CCA GGT TCT AGG CGT GCT GCT GTT TGG GGC G-3’) and T184A-R (5’-CAC GCC TAG AAC CTG GGC TGT GGA CCG CAC-3’) for EmAURKB^T184A^. Plasmids containing the wild-type or the mutant coding sequence of the kinases were then used for the transfection of human 293T cells.

In vitro kinase assay was performed as previously described [30]. Briefly, lysates of the 293T cells expressing HA-tagged EmAURKA, EmAURKB or their inactive mutants were incubated with anti-HA tag antibody conjugated-sepharose beads overnight at 4°C. The bead pellets were washed five times with cell lysis buffer, and then washed twice with kinase buffer (25 mM Tris-HCl at pH 7.5, 5 mM beta-glycerophosphate, 2 mM dithiothreitol, 0.1 mM Na3VO4, and 10 mM MgCl2). The pellets were then incubated with full length recombinant human Histone H3 as the substrate at 30°C for 30 minutes in kinase buffer supplemented with 200 μM ATP. For inhibitor assays, kinase reactions were performed in the presence or absence of 1 μM of MLN8237. Kinases and phosphorylated substrates were then detected by western blot using the anti-HA tag antibody and anti-phospho-Histone H3 Ser10 antibody, respectively. All primary antibodies were supplied by CST and used according to the manufacturer’s instructions.

### EdU labeling and immunofluorescence

For EdU labeling, *in vitro*-cultivated vesicles were treated with drugs or not, and then incubated with 50 μM of EdU for 4 hours and whole-mount prepared as described before [31]. Click-iT EdU Alexa Fluor 555 Imaging Kit (Life Technologies) was used for detection of EdU. Immunofluorescence was performed using the whole-mount prepared metacestode vesicles. Phosphorylation of Histone H3 was detected using anti-phospho-Histone H3 Ser10 or Ser28 antibodies (CST). For all immunofluorescence experiments, an Alexa 488-conjugated second antibody (Life Technologies) was used and DNA was counterstained with 4’, 6-diamidino-2-phenylindole (DAPI). For the quantification of EdU^+^, pH3^+^ or the multinucleated cells, at least 12 random microscopic fields from 4–6 vesicles were captured and the positive cells were counted. At least 3 labeling experiments were performed and analyzed.

### Data analysis and statistics

Data are shown as mean±SD or mean±SEM as indicated in the respective figure legend. Data within experiments were compared, and the significance was determined using two-tailed Student’s t-test.

## Results

### Identification of *E. multilocularis* Aurora kinases

By searching the *E*. *multilocularis* genome database, only two gene loci encoding members of Aurora kinase family, EmuJ_000891900 (annotated as serine/threonine protein kinase 12, STK12/Aurora kinase B) and EmuJ_001059700 (annotated as Aurora kinase A), were identified on chromosome 1 and 2, respectively. This is consistent with previous findings that mammals uniquely have three types of Aurora kinases (Aurora A, B and C), whereas other metazoans, including zebrafish, *Xenopus*, *Drosophila* and *C*. *elegans*, only Aurora A and Aurora B kinases are known [11]. Consistent with the annotation of *Echinococcus* genome project, we then named EmuJ_001059700 and EmuJ_000891900 as *EmAurka* and *EmAurkb*, respectively.

Informed by their genomic sequence, the entire reading frames of *EmAurka* and *EmAurkb* were PCR-amplified from metacestode cDNA preparations, and then cloned and sequenced. Our results show that the full length *EmAurka* coding sequence comprises 1050 bp, which is 198 bp longer than that determined by the genome project, resulting in a protein 66 amino acid residues longer. *EmAurka* gene spans a genomic region of 1.979 kb and comprises 7 exons, separated by 6 introns. Two additional short introns and one exon were identified in intron 1 of the predicted gene. Moreover, 57 of the 66 amino acid residues, which are absent in the protein determined by the genome project, are actually parts of the kinase domain of Aurora kinases (S1 Fig).

The coding sequence of *EmAurkb* we obtained is identical as determined by the *E*. *multilocularis* genome project. However, when we analyzed the sequence of the deduced protein, we found that the kinase domain is incomplete and the protein lacks N-terminal amino acids which are necessary for the cellular localization of Aurora kinases [32]. We then performed 5’ RACE and finally obtained an 873 bp-long reading frame encoding a protein 69 amino acids longer at the N-terminus of the deduced protein of EmuJ_000891900 (S2 Fig).

**Fig 1:**
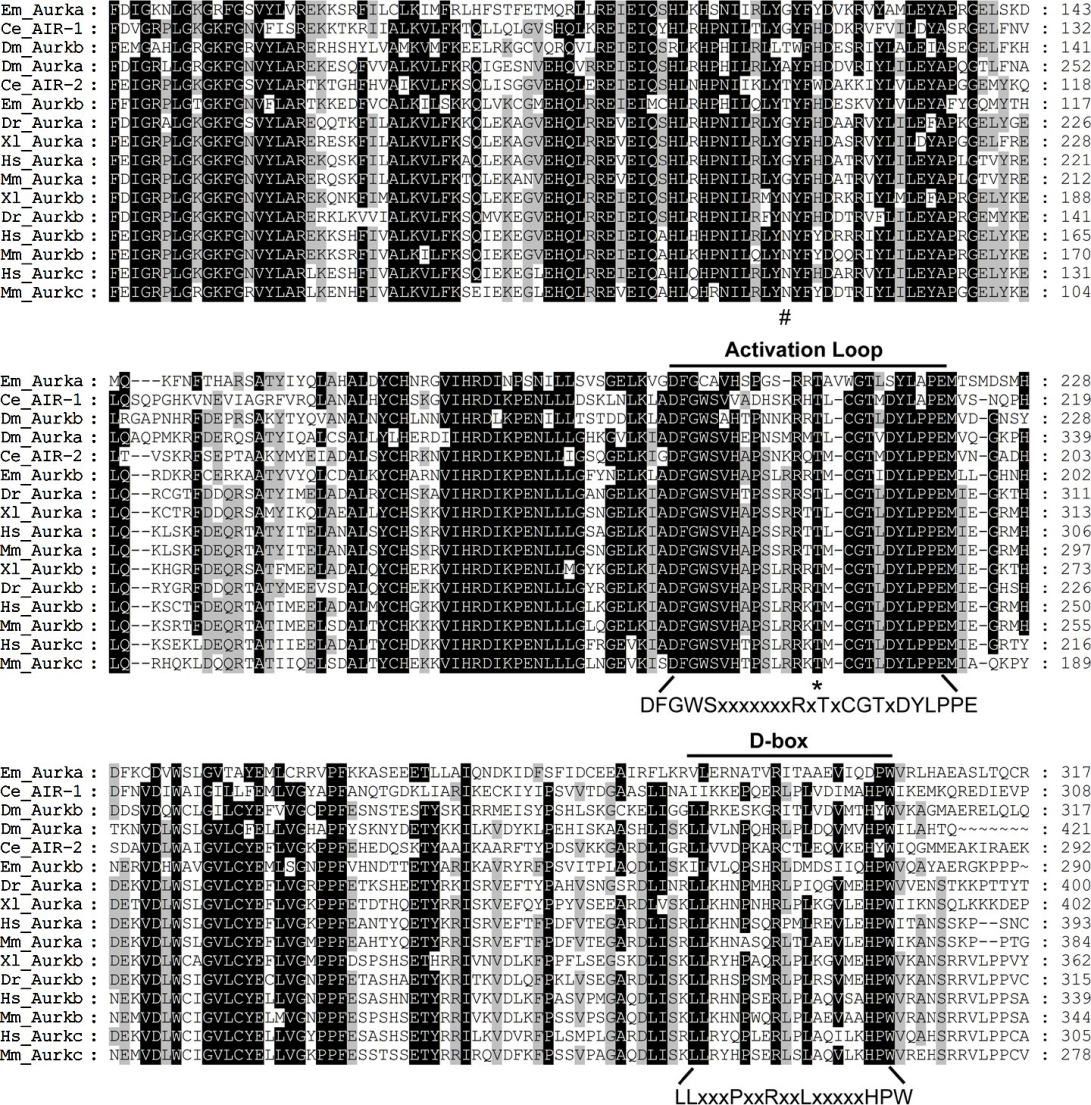
Alignment of *E. multilocularis* Aurora kinases with those from other organisms. Amino acid sequence of the catalytic kinase domain of EmAURKA and EmAURKB is aligned with that of other established Aurora kinase family members. Aurora signature motifs the “activation loop” (DFGWSxxxxxxxRxTxCGTxDYLPPE) and the destruction box, “D-box” (LLxxxPxxRxxLxxxxxHPW), are indicated. The conserved threonine residue within the RxT motif is indicated by “*”. The position of the residue corresponding to G198 in human Aurora A is indicated by “#”. Ce, *Caenorhabditis elegans*; Dm, *Drosophila melanogaster*; Xl, *Xenopus laevis*; Dr, *Danio rerio*; Mm, *Mus musculus*; Hs, *Homo sapiens*; Em, *Echinococcus multilocularis*.

Comparison of their protein sequences with the characterized Aurora kinases from other organisms shows that *E*. *multilocularis* Aurora homologs contain the highly conserved catalytic kinase domain, including the Aurora signature motif within the activation loop and the destruction box (D-box) near the C-terminus (Fig 1). In mammals, the full activation of Aurora kinase and proper organization of activation loop requires phosphorylation on the threonine residue within the conserved RxT motif (e.g. Thr288 in human Aurora A and Thr232 in human Aurora B, respectively) [7], and this residue is also conserved in both of the *E*. *multilocularis* Aurora kinases (Thr208 in EmAURKA and Thr184 in EmAURKB, respectively). Although EmAURKA and EmAURKB share comparatively lower homologies with each other in the kinase domain (41% identical residues), they display more significant homologies to their mammalian homologues (49% identical residues between EmAURKA and human Aurora A; 61% identical residues between EmAURKB and human Aurora B).

It has been demonstrated that all Aurora A kinases in model organisms share a common feature in that the amino acid sequence corresponding to human Aurora A residue 198 is glycine or alanine, which has a short and hydrophobic side chain. On the contrary, all Aurora B kinases share an amino acid with a long and hydrophilic side chain such as asparagine or threonine at this position [32–35]. Consistent with that, the residue corresponding to human Aurora A G198 is glycine in EmAURKA and threonine in EmAURKB, receptively (Fig 1).

### Expression of Aurora kinases in *E. multilocularis* larvae

Given that the expression and activity of Aurora A and Aurora B increase from late G2 through the M phase in mammalian cells [10, 35] and that the germinative cells represent the only proliferative cells in *E*. *multilocularis*, we speculated that *EmAurka* and *EmAurkb* are predominantly expressed in the germinative cells that actively proliferate throughout the whole developmental stages of the parasite. We then analyzed their expressions in four larval stages of *E*. *multilocularis*: mature protoscoleces, developing protoscoleces, protoscoleces undergoing microcyst formation and metacestode vesicles.

**Fig 2:**
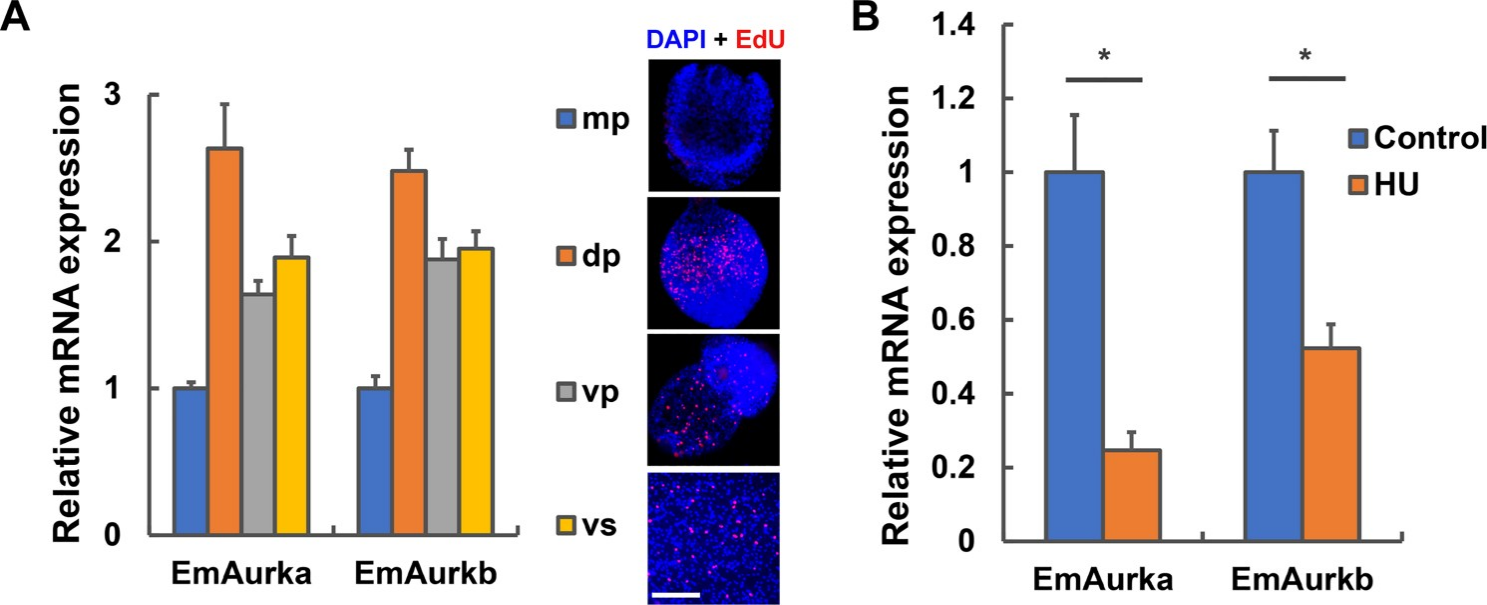
Expression of EmAurka and EmAurkb in *E. multilocularis* larvae. (A) Relative mRNA levels of *EmAurka* and *EmAurkb* in the mature protoscoleces (mp), developing protoscoleces (dp), protoscoleces undergoing vesicle formation (vp) and the metacestode vesicles (vs). Representative images of the proliferating germinative cells labeled with EdU (red) and counterstained with DAPI (blue) for the different developmental stages are shown on the right. Bar = 100 μm. Data are shown as mean ± SEM of three experiments. (B) mRNA levels of *EmAurka* and *EmAurkb* in the *in vitro*-cultivated metacestode vesicles treated with hydroxyurea (HU) or not (control) for 7 days. Data are shown as mean ± SEM of three separate experiments. * (*P* < 0.05, Student’s t-test).

As shown in Fig 2A, mRNA levels of both *EmAurka* and *EmAurkb* were much lower in the mature protoscoleces compared to the other three developmental stages, and the most prominent expression was observed in the developing protoscoleces. Previous studies have shown that the germinative cells are abundant in the developing protoscoleces and actively proliferate, whereas in the mature protoscoleces these cells remain in a quiescent state or with slow cell-cycle kinetics [5]. The protoscoleces undergoing microcyst formation and the metacestode vesicles also have a large population of proliferating germinative cells. These results may suggest that *EmAurka* and *EmAurkb* are specifically expressed in the proliferating germinative cells. We then analyzed their mRNA levels in metacestode vesicles treated with hydroxyurea, which could specifically deplete the germinative cell populations in *E*. *multilocularis* [5]. As expected, mRNA expression of both Aurora kinases was greatly downregulated after hydroxyurea treatment (Fig 2B). These results may suggest that *EmAurka* and *EmAurkb* are actively expressed in the germinative cells of *E*. *multilocularis*.

### EmAURKA and EmAURKB exhibit kinase activity in vitro

**Fig 3:**
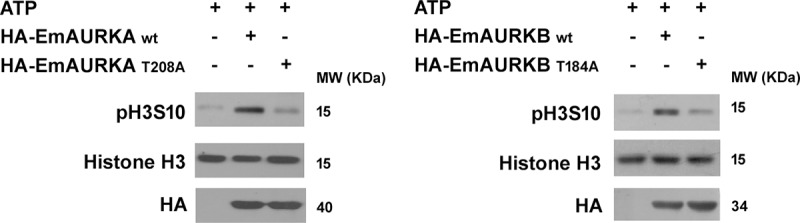
EmAURKA and EmAURKB display in vitro kinase activity. Lysates of human 293T cell expressing HA-tagged wild type EmAURKA, EmAURKB or their mutants EmAURKA^T208A^ and EmAURKA^T184A^ were immunoprecipitated by the anti-HA antibody. In vitro kinase assay was performed using recombinant human Histone H3 as the substrate. Phosphorylation of the substrate was detected by the anti-phospho-Histone H3 (Ser10) antibody.

Histone H3 is one of the most important substrates of Aurora B during mitosis and it can be phosphorylated by Aurora A *in vitro* [36, 37]. To explore if EmAURKA and EmAURKB possess enzymatical activities, we performed in vitro kinase assay using Histone H3 as the substrate. As shown in Fig 3, both EmAURKA and EmAURKB could phosphorylate human Histone H3. The kinase activity of Aurora A and B is regulated by phosphorylation of the threonine in the RxT motif within the activation loop, and mutations of this residue eliminate the kinase activity almost completely [38, 39]. We then generated inactive forms of both kinases by substituting alanine for threonine (T208A for EmAURKA and T184A for EmAURKB, respectively), and found that EmAURKA^T208A^ and EmAURKB^T184A^ had greatly reduced in vitro kinase activity. These results demonstrate that *E*. *multilocularis* Aurora kinases possess enzymatical activities.

### MLN8237 inhibits the activities of *E. multilocularis* Aurora kinases

Considering the similarities in the Aurora kinase domain shared by *E*. *multilocularis* and human and the evidence that the inhibitors originally designed to target human AURKs also have impact on the AURK homologues in invertebrates [40–42], we then tested the effects of various AURK inhibitors on *E*. *multilocularis*.

**Fig 4:**
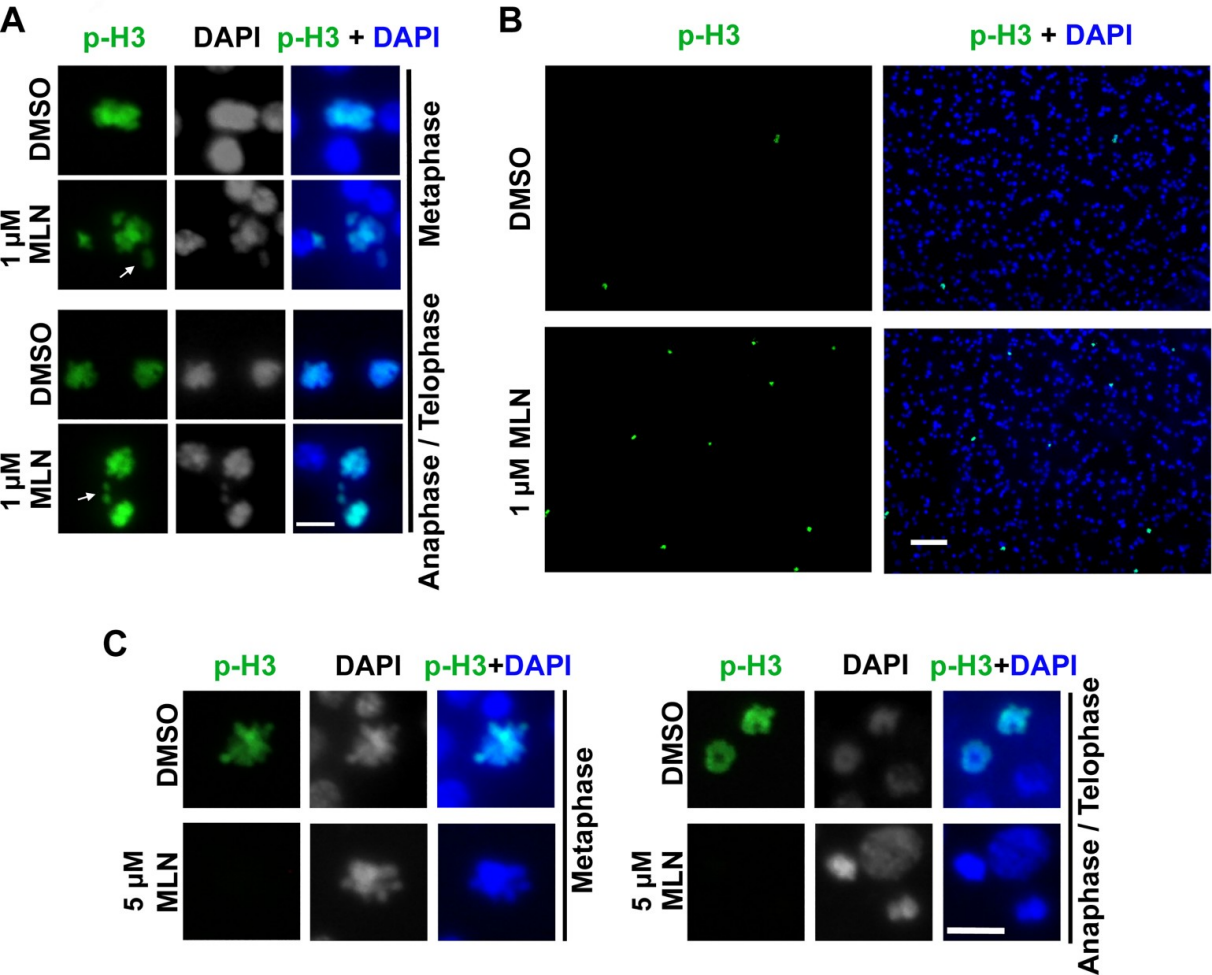
MLN8237 inhibits the activity of Aurora kinases in the mitotic germinative cells of *E. multilocularis*. (A) Low concentration of MLN8237 causes chromosomal defects in the mitotic germinative cells. Metacestode vesicles were treated with 1 μM of MLN8237 (MLN) or DMSO for 24h. Blue: DAPI. Green: phospho-Histone H3 (Ser10). Arrows indicate the misaligned or lagging chromosomes during metaphase and anaphase / telophase. Bar = 5 μm. (B) Low concentration of MLN8237 causes increased pSer10-Histone H3 positive cells. Metacestode vesicles were treated with 1 μM of MLN8237 or DMSO for 24h and then administrated to immunofluorescence using anti-phospho-Histone H3 (Ser10) antibody (green). DAPI was used for nuclei staining (blue). Bar = 50 μm. (C) High concentration of MLN8237 reduces the phosphorylation of Histone H3 at Ser10 in the mitotic germinative cells. Vesicles were treated with 5 μM of MLN8237 for 24 h and immunofluorescence was performed using anti-phospho-Histone H3 (Ser10) antibody (green) and DAPI for nuclei staining (blue). Bar = 5 μm.

We found that MLN8237, a potent inhibitor for human Aurora A which can also inhibit Aurora B activity at higher concentrations [37, 43–45], reduced the in vitro kinase activity of EmAURKA and EmAURKB (S3 Fig). We then treated *in vitro*-cultivated metacestode vesicles with different concentrations of MLN8237. As shown in Fig 4A, after the treatment of 1 μM MLN8237 for 24 h, about 20% of the mitotic cells (21.8 ±1.9%, three separate experiments) displayed misaligned or lagging chromosomes, which is indicative of Aurora A inhibition [46, 47].

Although Aurora A can phosphorylate Histone H3 *in vitro*, phosphorylation of Histone H3 during mitosis is mainly regulated by Aurora B rather than Aurora A *in vivo* [35–37]. We treated metacestode vesicles with 1 μM of MLN8237 for 24 h, and found no obvious changes in the level of phosphorylation of Histone H3 in the germinative mitotic cells, suggesting unimpaired activity of Aurora B (Fig 4A). However, we observed that the number of pSer10-Histone H3 positive cells was increased about 3–4 folds after the treatment of MLN8237 (3.8 ± 1.5 ‰ and 13.7 ± 4.0 ‰ of total cells for the control and MLN8237-treated groups, respectively) (Fig 4B), suggesting an elevated proportion of cells at G2/M phase, a phenotype caused by specific inhibition of Aurora A without affecting Aurora B activity [37, 48, 49]. These results indicate that MLN8237 can inhibit the activity of Aurora A in the germinative cells of *E*. *multilocularis*.

Aurora B phosphorylates Histone H3 at Ser10 and Ser28 in the N-terminal tail during mitosis, and phosphorylation of Histone H3 is considered as a robust and reproducible cellular readout for Aurora B kinase activity [37]. *E*. *multilocularis* Histone H3 exhibits significant homology to their mammalian relatives, and the two phosphorylation sites are also well conserved (S4A Fig). Our results indicate that phosphorylations of Histone H3 at Ser10 and Ser28 were greatly decreased in the mitotic germinative cells upon the treatment of 5 μM MLN8237 for 24 h (Fig 4C and S4B Fig), suggesting that MLN8237 can also inhibit Aurora B activity in *E*. *multilocularis*.

We also treated metacestode vesicles with nocodazole, a compound capable of arresting cells at G2/M by inhibiting microtubule polymerization [50, 51], to allow mitotic cells to accumulate. We found that pSer10-Histone H3 positive cells were apparently induced upon the treatment of nocodazole, whereas they were still hardly detected in the vesicles co-incubated with 5 μM of MLN8237 (S5 Fig). These data suggest that MLN8237-induced inhibition of Aurora B impairs the phosphorylation of Histone H3 in the mitotic cells of *E*. *multilocularis*.

Altogether, these results suggest that MLN8237 can inhibit the activities of both Aurora kinases in *E*. *multilocularis*, which may in turn cause severe impact on the mitotic progression of the germinative cells in *E*. *multilocularis*.

### Inhibition of *E. multilocularis* Aurora kinases impairs germinative cell maintenance

**Fig 5:**
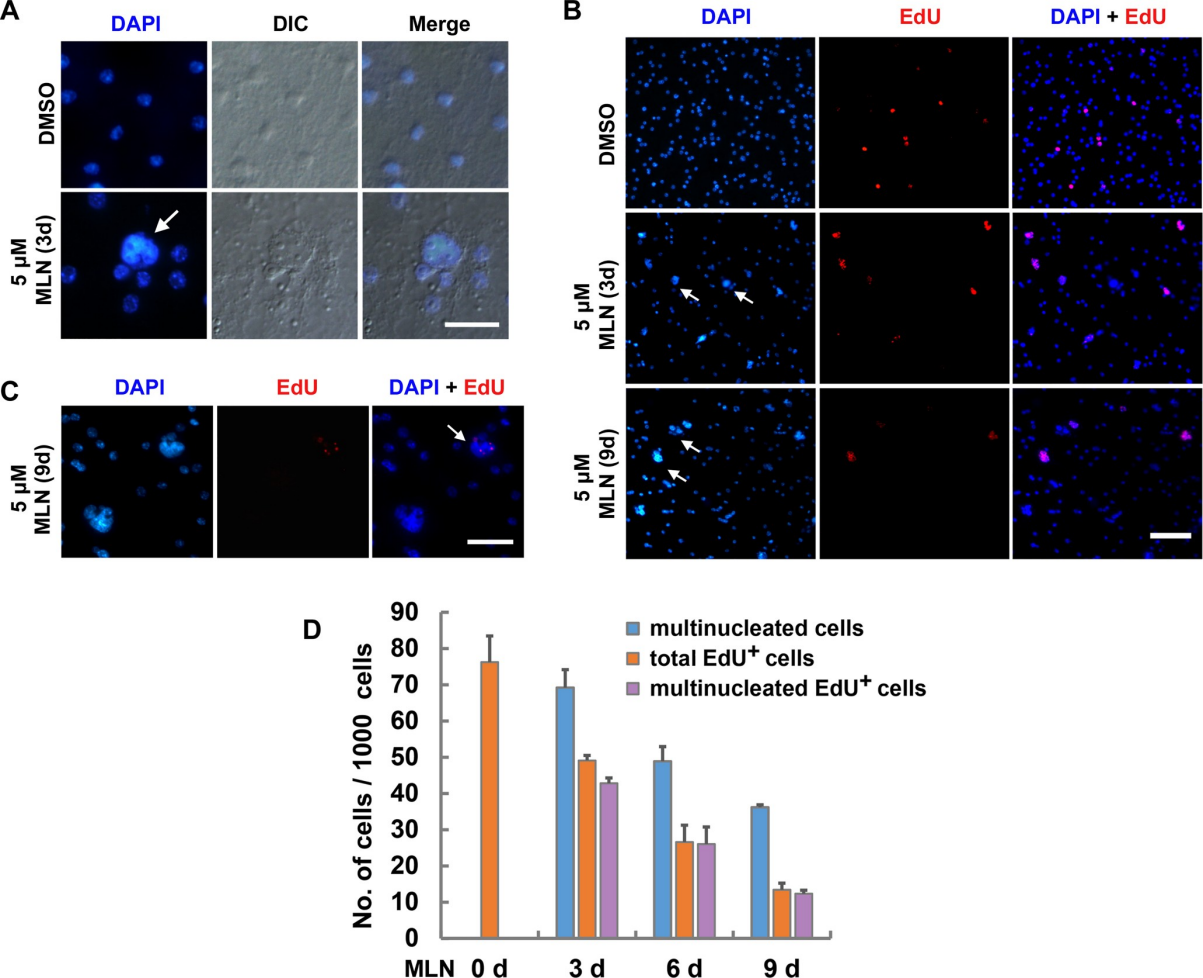
MLN8237 induces multinucleated germinative cells in *E. multilocularis*. (A) Representative images of multinucleated cells. Vesicles were treated with 5 μM MLN8237 (MLN) or DMSO for 3 days and stained with DAPI. Arrow indicates a multinucleated cell. Bar = 20 μm. (B) MLN8237 induces multinucleation of the germinative cells that can be labeled with EdU. Vesicles were treated with DMSO or 5 μM MLN8237 for the indicated time and then administrated to 4-hour pulse of EdU (red). DAPI was used for nuclei staining (blue). Arrows indicate the multinucleated cells. Bar = 50 μm. (C) Enlarged view of the multinucleated cells upon the treatment of 5 μM MLN8237 for 9 days. Arrow indicates a multinucleated cell with impaired EdU (red) labeling detected by long time of exposure. Bar = 20 μm. (D) Quantification of the multinucleated cells, total EdU^+^ cells and multinucleated EdU^+^ cells in the vesicles treated with 5 μM MLN8237 (MLN) for the indicated time. Data indicate the number of each type of cells per 1000 total cells and are shown as mean ± SD of three separate experiments. Note that control group (0 day) display neither multinucleated cells nor multinucleated EdU^+^ cells but only EdU^+^ cells. Ratio of the multinucleated EdU^+^ cells to total multinucleated cells or to total EdU^+^ cells is shown in S6 Fig.

We found that 5 μM of MLN8237 also induced multinucleated cells in the germinal layer of the vesicles, a hallmark for Aurora B inhibition [49, 52, 53]. Formation of multinuclei or a single large nucleus could be observed as early as day 1 during treatment, and the number of multinucleated cells greatly increased on day 3 (Fig 5A and S6A Fig). The phenotype of multinucleation should only be existing in the germinative cells, the only cells capable of proliferation and division in *E*. *multilocularis*. These multinucleated cells could enter and exit mitosis but failed in completing cytokinesis due to the inhibition of Aurora B.

We then incubated MLN8237-treated vesicles with EdU, an analogue of thymidine which can be incorporated by the germinative cells at S phase [5, 27], and found that over 60% of the multinucleated cells were EdU^+^ after 3 days of MLN8237 treatment. On the other hand, almost all of the EdU^+^ cells exhibited multinucleation phenotype during the whole time of treatment (Fig 5B and 5D and S6B Fig), suggesting that MLN8237 displays specific effects on the germinative cells.

After 9 days of treatment, the multinucleated cells exhibited apparently impaired ability of incorporating EdU, and about 70% of the multinucleated cells were not labeled with EdU (Fig 5C and 5D and S6B and S6C Fig), probably because these germinative cells displayed a delayed cell cycle or could no longer continue to go through the cell cycle. In addition, after 6 and 9 days of treatment with 5 μM MLN8237, we could hardly detect cells exhibiting the M-phase typical DAPI-staining of condensed chromosomes, suggestive of severe mitotic errors.

We also tested the effects of MK-5108 (Aurora A-selective inhibitor), AZD1152-HQPA (Aurora B-selective inhibitor) and MLN8054 (the predecessor of MLN8237) [49, 54, 55], and found no severe defects in the germinative cells upon the treatment of MK-5108 or AZD1152-HQPA, e.g. chromosome misalignment, abnormal multinucleated cells and impaired phosphorylation of Histone H3. However, MLN8054 exhibited very similar effects as MLN8237, despite higher concentrations needed to give rise to the phenotypes of Aurora A/B inhibition (S7 Fig), confirming our observations about the effects of MLN8237 on the germinative cells of *E*. *multilocularis*.

**Fig 6:**
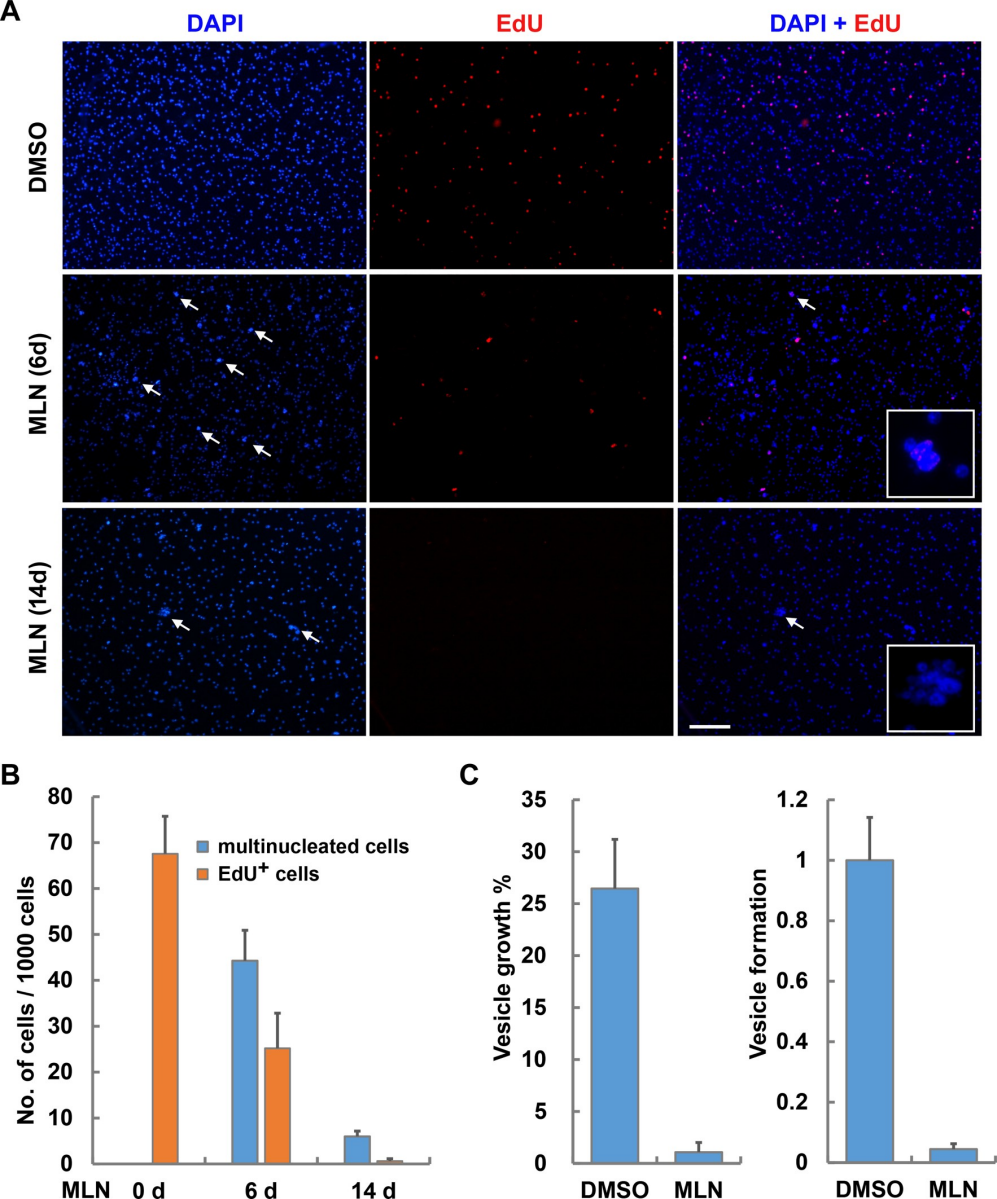
Inhibition of Aurora kinases by MLN8237 impairs germinative cell maintenance and larval growth of *E. multilocularis*. (A) Long-term MLN8237 treatment eliminates germinative cell populations. Vesicles were treated with 5 μM MLN8237 (MLN) for 6 days or 14 days followed by 4-h pulse of EdU (red). Note the greatly decreased number of the multinucleated cells indicated by arrows on day 14 compared to that on day 6. Inserts are shown as the magnified view of the multinucleated cells. Bar = 100 μm. (B) Quantification of the multinucleated cells and EdU^+^ cells in the vesicles treated with 5 μM of MLN8237 for 6 days and 14 days. Data indicate the number of each type of cells per 1000 total cells and are shown as mean ± SD of three separate experiments. (C) Effects of MLN8237 on the larval growth and development of *E*. *multilocularis*. Vesicles or protoscoleces were cultivated in conditioned medium containing DMSO or 5 μM MLN8237. Vesicle growth (left) and initial process of vesicle formation from protoscoleces (right) were analyzed after 21 days and 14 days of treatment, respectively. Vesicle formation of control group is set to 1. Data are shown as mean ± SD of three experiments.

We then prolonged the time of MLN8237 treatment to 14 days. Strikingly, the number of the multinucleated cells was decreased to only about 0.5% of total cells, and EdU was hardly detected in the remaining multinucleated cells (Fig 6A and 6B). We also allowed vesicles to recover for 3 days in drug-free media after 14 days of treatment and then performed EdU labeling, and EdU^+^ cells were still hardly detected (S8 Fig). These results indicate that inhibition of the Aurora kinases eliminates the germinative cells in *E*. *multilocularis* metacestode vesicles.

When exposed to MLN8237 for 21 days, the *in vitro*-cultivated metacestode vesicles did not display structural disintegration or collapse, however, they could no longer grow. In addition, the initial process of vesicle formation from protoscoleces was also greatly inhibited by MLN8237 (Fig 6C). These results suggest that Aurora inhibition impairs the maintenance of germinative cell populations, which in turn leads to the impaired larval growth and development of *E*. *multilocularis*.

## Discussion

The current chemotherapy for human AE mainly relies on benzimidazoles, which do not, however, act parasiticidally *in vivo*. It has been shown that benzimidazoles have limited effects on killing the germinative cells of *E*. *multilocularis*, probably because these cells express specific β-tubulin isoforms which are resistant to inhibition by benzimidazoles [6]. The stem cell-like germinative cells drive the larval growth and development of *E*. *multilocularis* within the hosts, and are considered the main cause of disease recurrence. Therefore, germinative cells are suggested as crucial targets for anti-echinococcosis drug development [6]. Recently, multiple lines of evidence have shown that drugs which impact the function of the germinative cells could efficiently inhibit, at least *in vitro*, the growth of *E*. *multilocularis* larvae [27, 29, 56].

As the only proliferative cells in *E*. *multilocularis*, germinative cells differentiate into other types of cells and also undergo extensive proliferation and self-renewal, maintaining their populations in the parasite [5]. Cell proliferation needs accurate control of the cell division cycle, which includes interphase, mitotic phase, and cytokinesis in eukaryotes. In the case of human cancer, therapeutic strategies have been proposed for targeting cell division, and targeting the mitotic progression is considered to offer possibilities for killing cancer cells [57]. Strikingly, these strategies have become referential for anti-human protozoan drug discovery, and an increasing number of investigations have focused on the regulatory components of protozoan cell division [22–23]. As concerning *E*. *multilocularis*, Schubert et al. have recently shown that inhibition of the Polo-like kinase EmPlk1 by the compound BI2536 could prevent the larval growth of the parasite, possibly by specifically inducing mitotic arrest and cell death of the germinative cells [29].

The conserved Aurora family of protein kinases are crucial regulators of essential processes ranging from mitotic entry to cytokinesis. Dysfunction of Aurora kinases causes aberrant mitosis, aneuploid or chromosome instability, which may in turn lead to cell senescence and death [7, 10]. We herein describe the identification of two druggable Aurora homologues in *E*. *multilocularis*, which play vital roles in regulating germinative cell mitosis. We show that both Aurora kinases, EmAURKA and EmAURKB are structurally homologous to the mammalian orthologs and contain conserved residues at corresponding positions which are essential for the kinase activity (Figs 1 and 3).

Activated Aurora A phosphorylates, and thus activates, its substrate PLK1 at T210 during mitosis in mammalian cells [7, 35]. The *E*. *multilocularis* PLK1 homologue, EmPLK1, has been identified and the corresponding threonine residue (T179) is also conserved in EmPLK1 [29], implicating a similar activation mechanism in *E*. *multilocularis* mitotic germinative cells. The transcription factor p53 is another prominent substrate involved in the checkpoint and apoptotic responses regulated by Aurora kinases [7, 10]. We have previously characterized the p53 homologue Emp53 in *E*. *multilocularis* [58]. Our recent studies found that Emp53 has two putative Aurora regulated-serine phosphorylation sites within the RxS motifs. It would be tempting to explore the functional significance of Aurora-p53 interactions in the regulation of *E*. *multilocularis* germinative cells in our future work.

Our data demonstrate that *EmAurka* and *EmAurkb* are highly expressed in the larval stages which have a large population of germinative cells extensively proliferating (Fig 2A). In addition, the expression of both kinases is significantly decreased upon the specific depletion of germinative cell populations by hydroxyurea treatment (Fig 2B). Since the germinative cells are the only cells capable of proliferation and division in *E*. *multilocularis*, these results may suggest that *E*. *multilocularis* Aurora kinases are specifically expressed in the germinative cells.

Due to their crucial roles in regulating mitosis, Aurora kinases have emerged as attractive drug targets in cancer and have become the focus of intense drug discovery efforts [14, 15]. A number of Aurora kinase inhibitors are developed, and some of them have been shown to also inhibit the Aurora kinases in protozoan, *C*. *elegans* and *Drosophila* [19–22, 40–42]. Our findings indicate that MLN8237 and its predecessor MLN8054 display significant effects on the mitotic germinative cells of *E*. *multilocularis* metacestode larvae (Fig 4, Fig 5 and S7 Fig). MLN8237 was initially designed as an Aurora A inhibitor, but also inhibits Aurora B at higher concentrations. In proliferation inhibition studies, MLN8237 exhibits activities against human tumor cell lines with IC50 values ranging from 15 to 469 nM [43]. In phenotype-based cellular assays, the considerable concentrations for selective inhibition of Aurora A range from 0.1 to 0.5 μM depending on the cell lines used, whereas 1 μM and higher concentrations of MLN8237 are necessary for Aurora B inhibition [37, 43, 45, 59]. Studies using animal models showed that the corresponding plasma concentration needed to inhibit Aurora A *in vivo* is approximately 1 to 2 μM [43]. In this study, we found that 0.5 μM of MLN8237 could slightly inhibit Aurora A in the *in vitro*-cultivated *E*. *multilocularis* metacestode larvae, resulting in chromosome misalignment in a few of mitotic cells. Further experiments demonstrated phenotypes of Aurora A inhibition by using 1 μM of MLN8237, which caused severe chromosomal defects in the mitotic germinative cells and increased the proportion of cells at G2/M phase (Fig 4A and 4B).

Previous studies have shown that the specificity window for MLN8237 against Aurora A and B is narrow and it varies in different tumor cell lines, making it difficult to fully inhibit Aurora A without affecting Aurora B [37]. In *E*. *multilocularis*, although a 24 h-treatment of 1 μM MLN8237 gave rise to phenotypes of specific inhibition of Aurora A with little effects on Aurora B activity (Fig 4A and 4B), multinucleated cells could also be observed upon the treatment with 1 or 2 μM of MLN8237 for longer time in our studies. We therefore hypothesize that MLN8237 exhibits weak specificity for the inhibition of Aurora A/B in the germinative cells of *E*. *multilocularis*.

We show that the treatment with higher concentration of MLN8237 apparently gave rise to phenotypes of Aurora B inhibition in the germinative cells, e.g. impaired phosphorylation of Histone H3 (Fig 4C, S4 Fig and S5 Fig) and multinucleation (Fig 5A and S6A Fig). The EdU^+^ cells always exhibited multinuclei, suggestive of specific effects of MLN8237 on the mitotic germinative cells (Fig 5B and 5D and S6B Fig). Importantly, the numbers of EdU^+^ cells were gradually reduced since day 3 of the treatment, and almost all of the multinucleated cells were vanished after 14 days of treatment (Fig 5E, Fig 6A and 6B), suggestive of impaired viability of the germinative cells.

MLN8237 can inhibit the larval growth and development of *E*. *multilocularis* (Fig 6C). The metacestode vesicles could not grow but remained structurally intact upon the treatment of MLN8237 for weeks, similar to the vesicles treated with hydroxyurea or BI2536, both of which are considered to cause specific depletion of the germinative cell populations [5, 29].

Taken together, we propose that inhibition of *E*. *multilocularis* Aurora kinases by MLN8237 specifically causes germinative cell killing, resulting in impaired maintenance of the germinative cell populations and in turn larval growth inhibition of the parasite.

In conclusion, we herein present the Aurora kinases of *E*. *multilocularis* as promising druggable targets for eliminating germinative cells in the parasite. MLN8237 or other related Aurora inhibitors may serve as lead compounds for the development of novel drugs that target the mitotic progression of *Echinococcus* germinative cells and would be considered as alternative or complementary chemotherapeutics of benzimidazoles against human echinococcosis. On the other hand, our studies reveal Aurora kinases implicated in maintaining the germinative cell populations of *E*. *multilocularis*, offering a solid basis for further explorations of Aurora kinase-relevant mitotic regulators and mechanisms governing the unique stem cell system of the parasite in the future.

## Supporting information

S1 Fig Gene sequence and amino acid sequence of *EmAurka*. (A) Gene sequence of *EmAurka*. Coding sequence is shown in uppercase and the translational start / stop codons are shown in bold. The length of each intron and their partial sequence (italic lowercase) are given. Coding sequence identified in this study is indicated by single line “__”.
(B) Amino acid sequence of EmAURKA. Sequence corresponding to the catalytic kinase domain is shaded in grey. Amino acid sequence identified in this study is indicated by single line “__”.
(TIF)

S2 Fig Gene sequence and amino acid sequence of *EmAurkb*. (A) Gene sequence of *EmAurkb*. Coding sequence is shown in uppercase and the translational start / stop codons are shown in bold. The length of each intron and their partial sequence (italic lowercase) are given. Coding sequence identified in this study is indicated by single line “__”.
(B) Amino acid sequence of EmAURKB. Sequence corresponding to the catalytic kinase domain is shaded in grey. Amino acid sequence identified in this study is indicated by single line “__”.
(TIF)

S3 Fig MLN8237 reduces in vitro kinase activities of EmAURKA and EmAURKB. Recombinant HA-tagged EmAURKA and EmAURKB were immunoprecipitated from 293T cell lysates and then administrated to in vitro kinase assay using recombinant human Histone H3 as the substrate in the presence or absence of 1 μM of MLN8237 (MLN).
(TIF)

S4 Fig MLN8237 reduces the phosphorylation of Histone H3 on Ser28 in the mitotic germinative cells of *E. multilocularis*. (A) Amino acid sequence alignment of *E*. *multilocularis* Histone H3 (EmuJ_000579800) with those from other organisms. Residues corresponding to Ser10 and Ser28 of human Histone H3 are indicated by “*”. Hs, *Homo sapiens*; Mm, *Mus musculus*; Dm, *Drosophila melanogaster*; Ce, *Caenorhabditis elegans*; Em, *Echinococcus multilocularis*. (B) MLN8237 reduces Ser28 phosphorylation of Histone H3 in the mitotic germinative cells. Metacestode vesicles were treated with 5 μM of MLN8237 or DMSO for 24h. Phosphorylation of Histone H3 was detected by immunofluorescence using anti-phospho-Histone H3 (Ser28) antibody (green). DAPI was used for nuclei staining (blue). Bar = 5 μm.
(TIF)

S5 Fig MLN8237 impairs phosphorylation of Histone H3 in the mitotic germinative cells. *In vitro*-cultivated metacestode vesicles were treated with DMSO, or 0.5 μg/mL nocodazole alone (NOC), or nocodazole and 5 μM MLN8237 (MLN + NOC) for 24 h. Immunofluorescence was carried out to detect the phosphorylation of Histone H3 using anti-phospho-Histone H3 (Ser10) antibody (green). DAPI was used for nuclei staining (blue). Bar = 50 μm.
(TIF)

S6 Fig MLN8237 induces multinucleation in *E. multilocularis* metacestode vesicles (related to Fig 5). (A) Representative images of the *in vitro*-cultivated metacestode vesicles treated with DMSO or 5 μM MLN8237 (MLN) for the indicated time. Multinucleated cells were determined by DAPI staining. Arrows indicate the multinucleated cells. Bar = 100 μm. (B) Metacestode vesicles were treated with 5 μM MLN8237 for 3, 6 and 9 days. Ratio of the multinucleated (MN) EdU+ cells to total multinucleated cells or to total EdU+ cells at each timepoint is shown on the left and the right, respectively. Data are shown as mean ± SD of three separate experiments.
(TIF)

S7 Fig MLN8054 inhibits the activities of Aurora kinase in the mitotic germinative cells of *E. multilocularis*. (A) Low concentration of MLN8054 causes chromosomal defects in the mitotic germinative cells. Metacestode vesicles were treated with 5 μM of MLN8054 (MLN) or DMSO for 24h. Blue: DAPI. Green: phospho-Histone H3 (Ser10). Arrows indicate the misaligned or lagging chromosomes during metaphase and anaphase / telophase. Bar = 5 μm. (B) High concentration of MLN8054 induces multinucleated germinative cells. *In vitro*-cultivated metacestode vesicles were treated with DMSO or 20 μM MLN8054 (MLN) for 9 days followed by 4-hour pulse of EdU (red). Arrows indicate the multinucleated cells. Bar = 25 μm.
(TIF)

S8 Fig Long-term MLN8237 effects on *E. multilocularis* germinative cells. Vesicles were treated with 5 μM MLN8237 for 14 days (MLN) or allowed to recover for 3 days in drug-free media after 14 days of treatment (MLN recovery), and then administrated to 4-hour pulse of EdU (red). DAPI was used for nuclei staining (blue). Inserts are shown as the magnified view of the multinucleated cells. Bar = 100 μm.
(TIF)
