# Supplementary figures and images for: Impairing the maintenance of germinative cells in Echinococcus multilocularis by targeting Aurora kinase
Source: PLoS Negl Trop Dis. 2019 May 16;13(5):e0007425. doi: 10.1371/journal.pntd.0007425 (PMC6541280; doi:10.1371/journal.pntd.0007425)

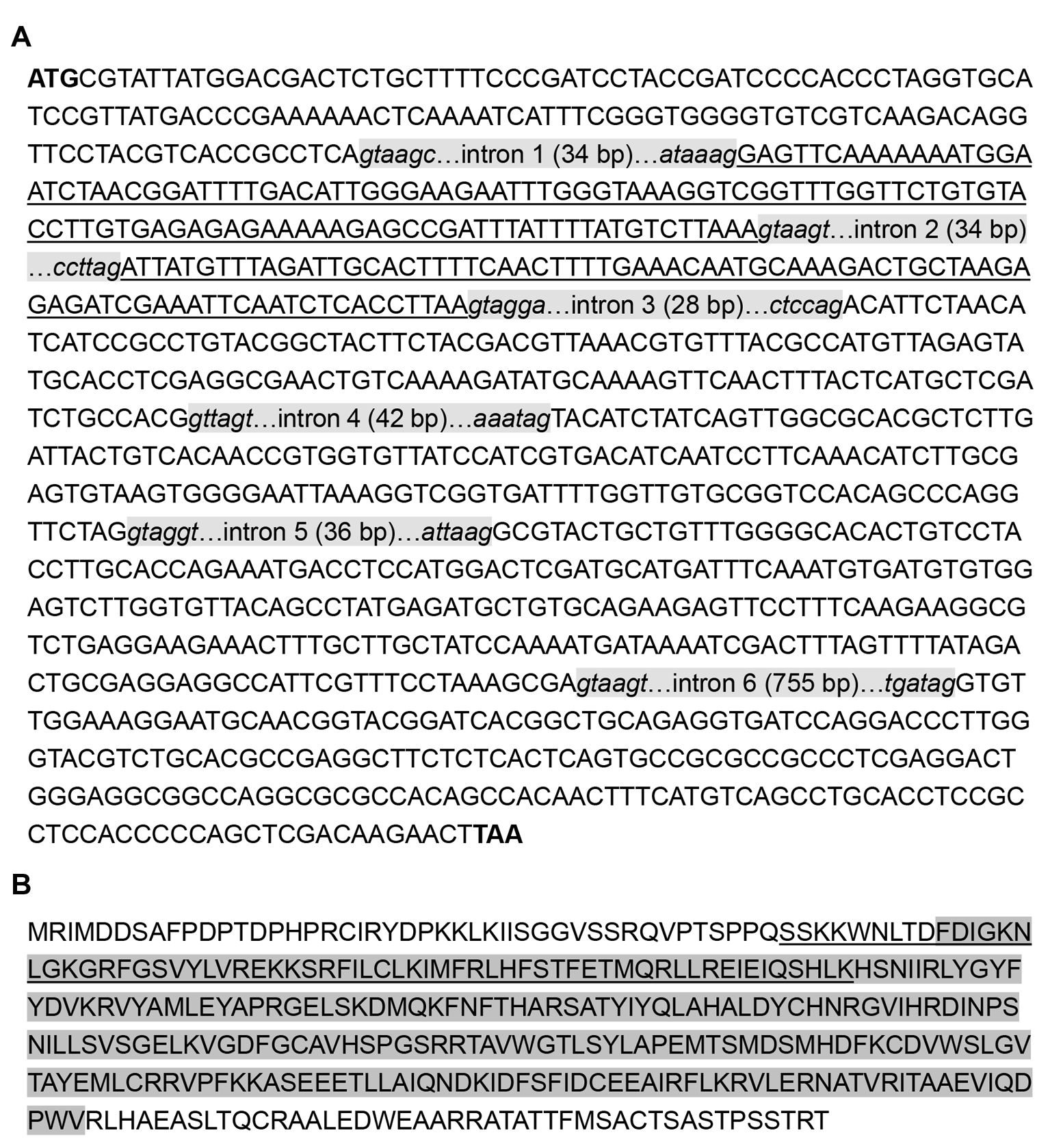

Supplement: S1 Fig — (A) Gene sequence of EmAurka. Coding sequence is shown in uppercase and the translational start / stop codons are shown in bold. The length of each intron and their partial sequence (italic lowercase) are given. Coding sequence identified in this study is indicated by single line “__”. (B) Amino acid sequence of EmAURKA. Sequence corresponding to the catalytic kinase domain is shaded in grey. Amino acid sequence identified in this study is indicated by single line “__”. (TIF) [file pntd.0007425.s001.tif]

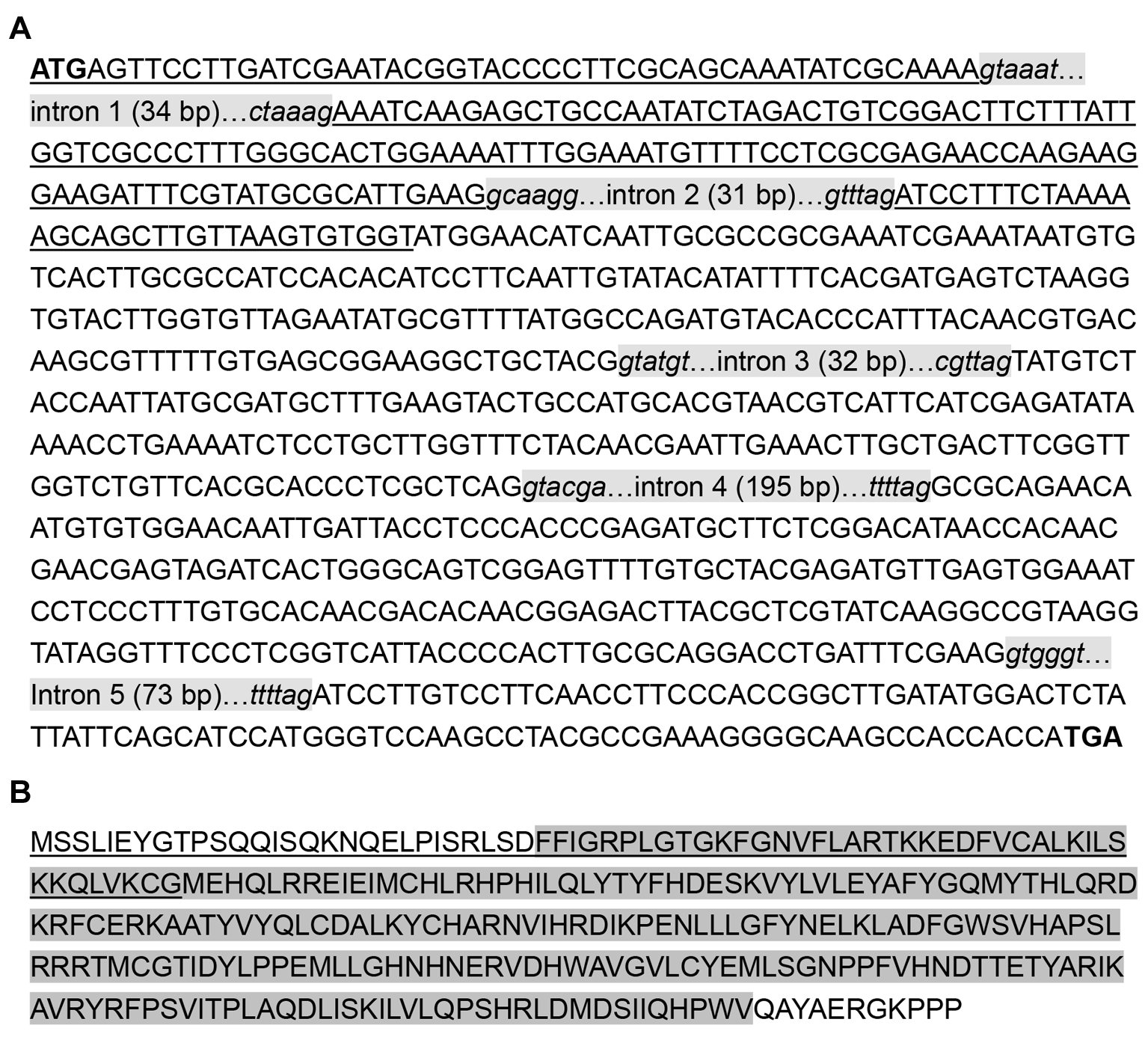

Supplement: S2 Fig — (A) Gene sequence of EmAurkb. Coding sequence is shown in uppercase and the translational start / stop codons are shown in bold. The length of each intron and their partial sequence (italic lowercase) are given. Coding sequence identified in this study is indicated by single line “__”. (B) Amino acid sequence of EmAURKB. Sequence corresponding to the catalytic kinase domain is shaded in grey. Amino acid sequence identified in this study is indicated by single line “__”. (TIF) [file pntd.0007425.s002.tif]

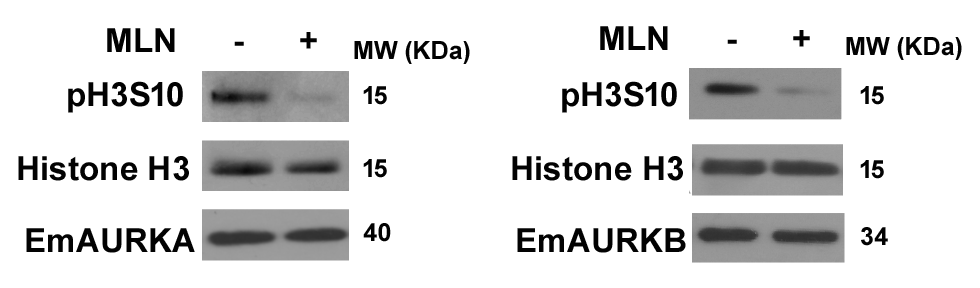

Supplement: S3 Fig — Recombinant HA-tagged EmAURKA and EmAURKB were immunoprecipitated from 293T cell lysates and then administrated to in vitro kinase assay using recombinant human Histone H3 as the substrate in the presence or absence of 1 μM of MLN8237 (MLN). (TIF) [file pntd.0007425.s003.tif]

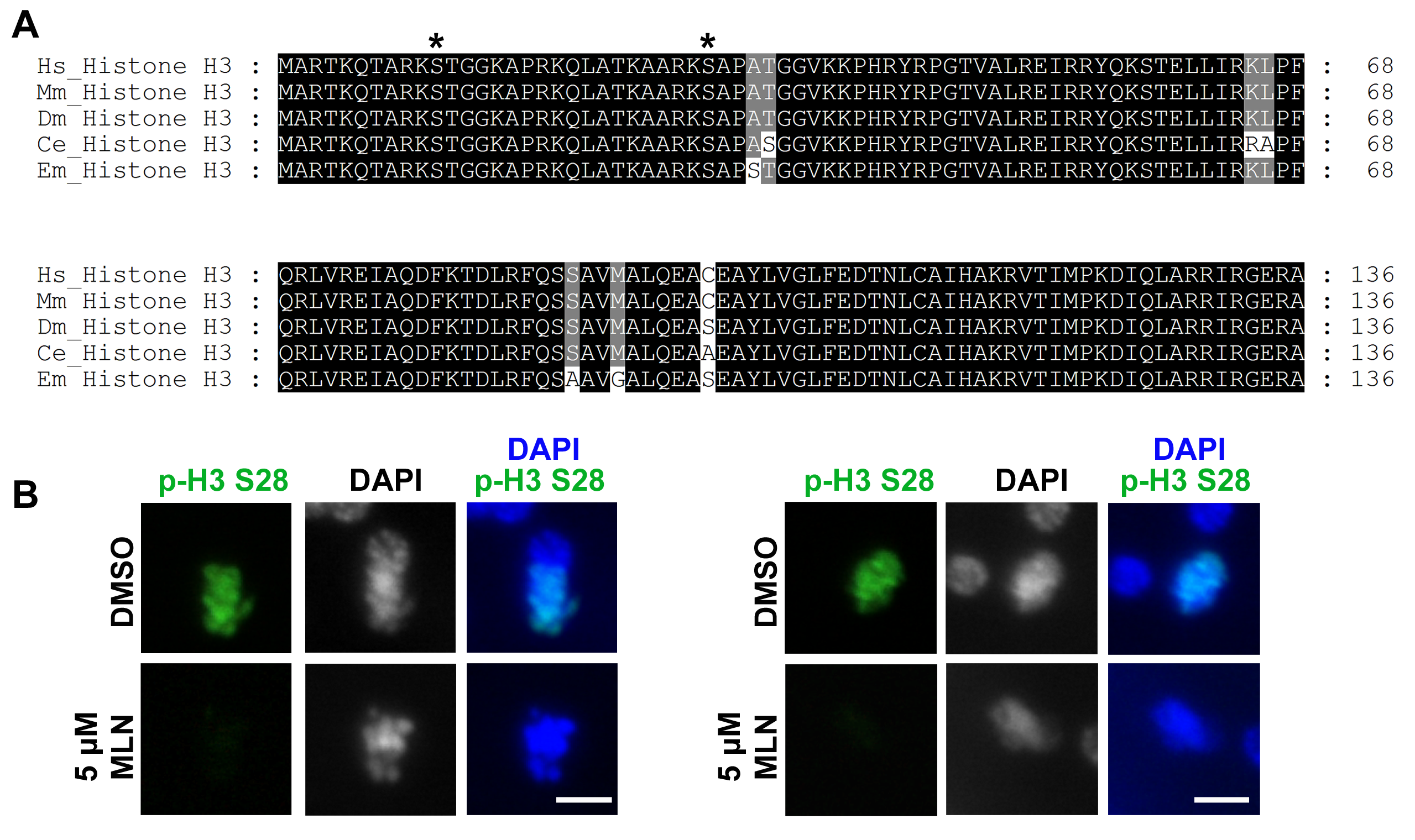

Supplement: S4 Fig — (A) Amino acid sequence alignment of E. multilocularis Histone H3 (EmuJ_000579800) with those from other organisms. Residues corresponding to Ser10 and Ser28 of human Histone H3 are indicated by “*”. Hs, Homo sapiens; Mm, Mus musculus; Dm, Drosophila melanogaster; Ce, Caenorhabditis elegans; Em, Echinococcus multilocularis. (B) MLN8237 reduces Ser28 phosphorylation of Histone H3 in the mitotic germinative cells. Metacestode vesicles were treated with 5 μM of MLN8237 or DMSO for 24h. Phosphorylation of Histone H3 was detected by immunofluorescence using anti-phospho-Histone H3 (Ser28) antibody (green). DAPI was used for nuclei staining (blue). Bar = 5 μm. (TIF) [file pntd.0007425.s004.tif]

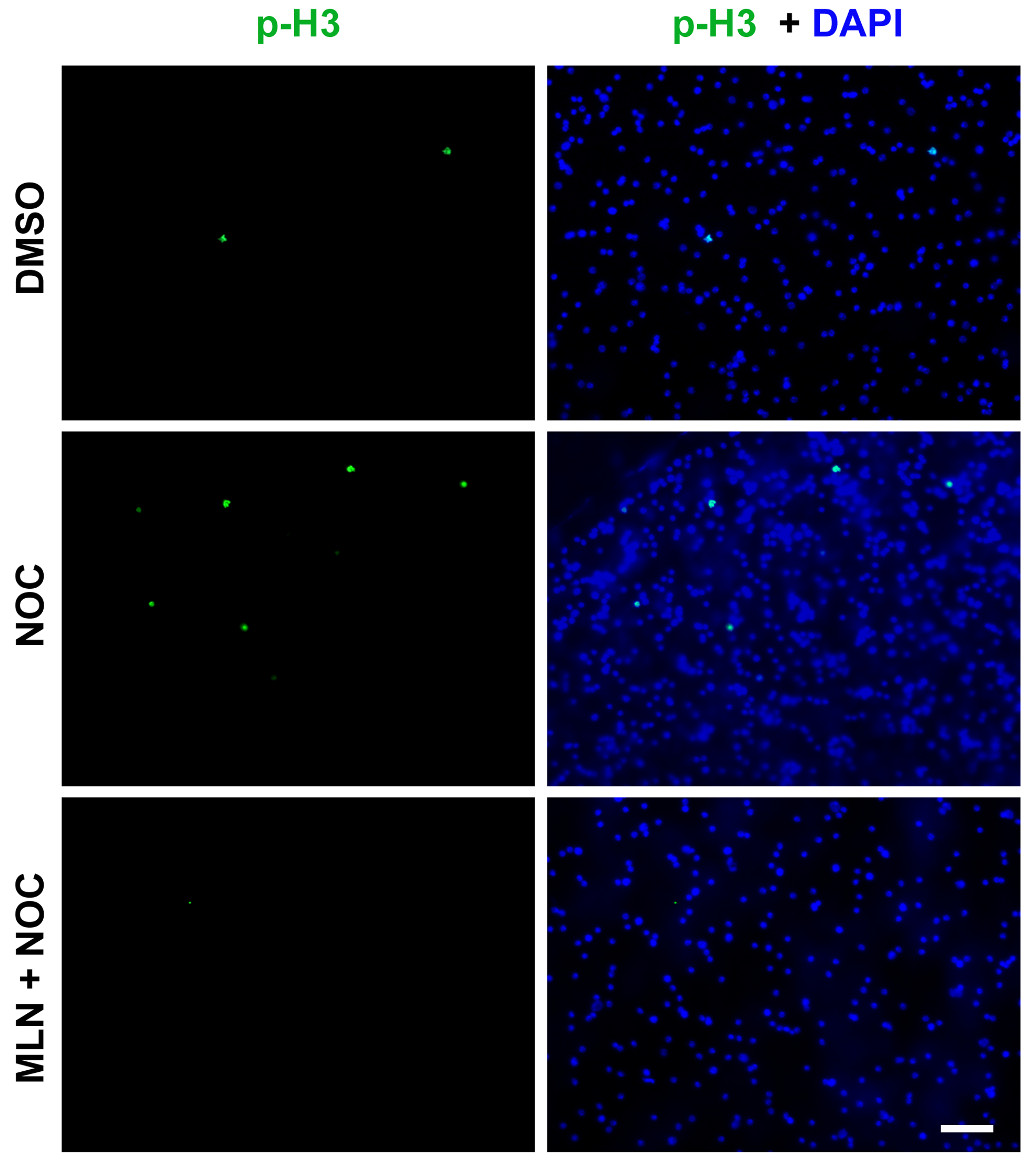

Supplement: S5 Fig — In vitro-cultivated metacestode vesicles were treated with DMSO, or 0.5 μg/mL nocodazole alone (NOC), or nocodazole and 5 μM MLN8237 (MLN + NOC) for 24 h. Immunofluorescence was carried out to detect the phosphorylation of Histone H3 using anti-phospho-Histone H3 (Ser10) antibody (green). DAPI was used for nuclei staining (blue). Bar = 50 μm. (TIF) [file pntd.0007425.s005.tif]

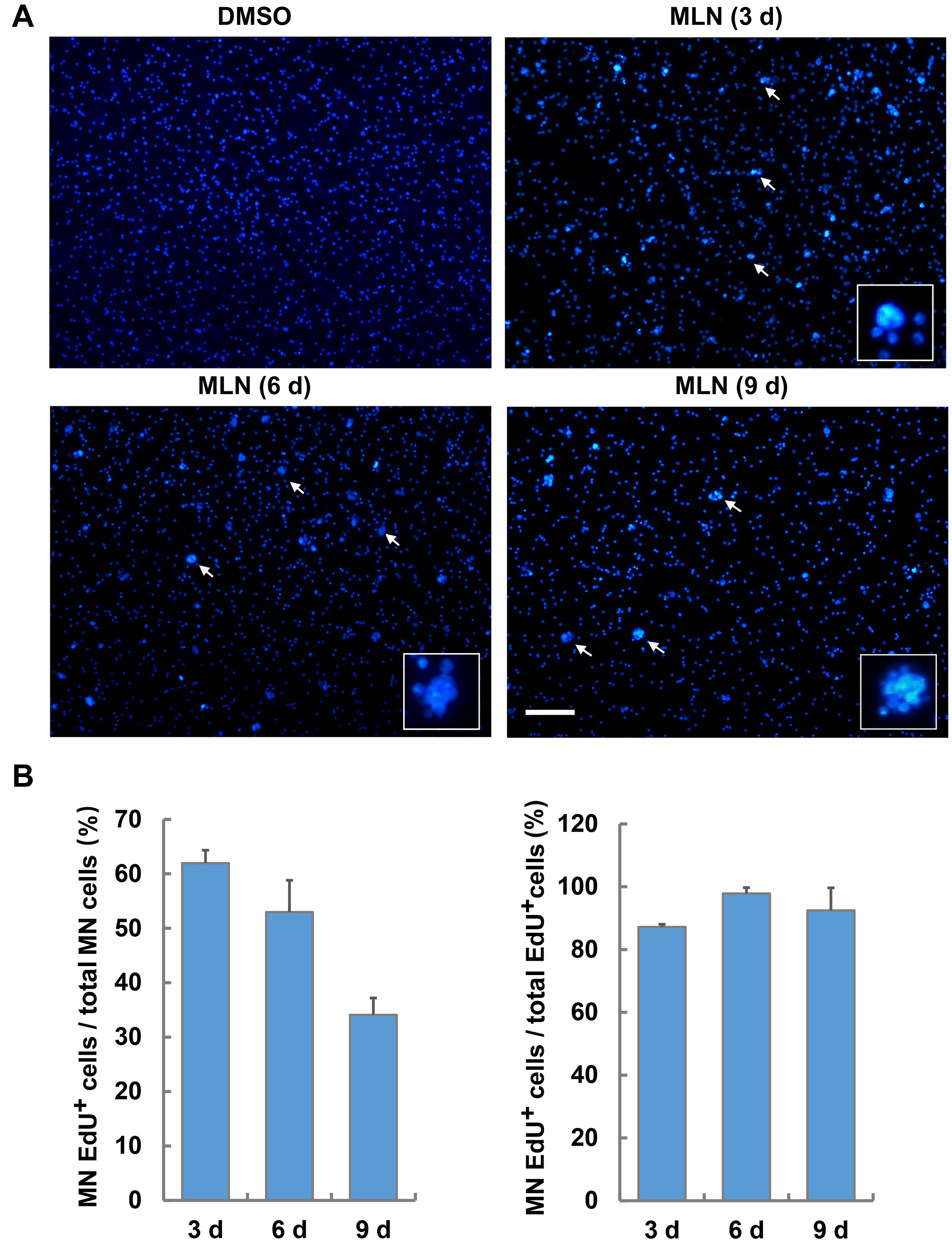

Supplement: S6 Fig — (A) Representative images of the in vitro-cultivated metacestode vesicles treated with DMSO or 5 μM MLN8237 (MLN) for the indicated time. Multinucleated cells were determined by DAPI staining. Arrows indicate the multinucleated cells. Bar = 100 μm. (B) Metacestode vesicles were treated with 5 μM MLN8237 for 3, 6 and 9 days. Ratio of the multinucleated (MN) EdU+ cells to total multinucleated cells or to total EdU+ cells at each timepoint is shown on the left and the right, respectively. Data are shown as mean ± SD of three separate experiments. (TIF) [file pntd.0007425.s006.tif]

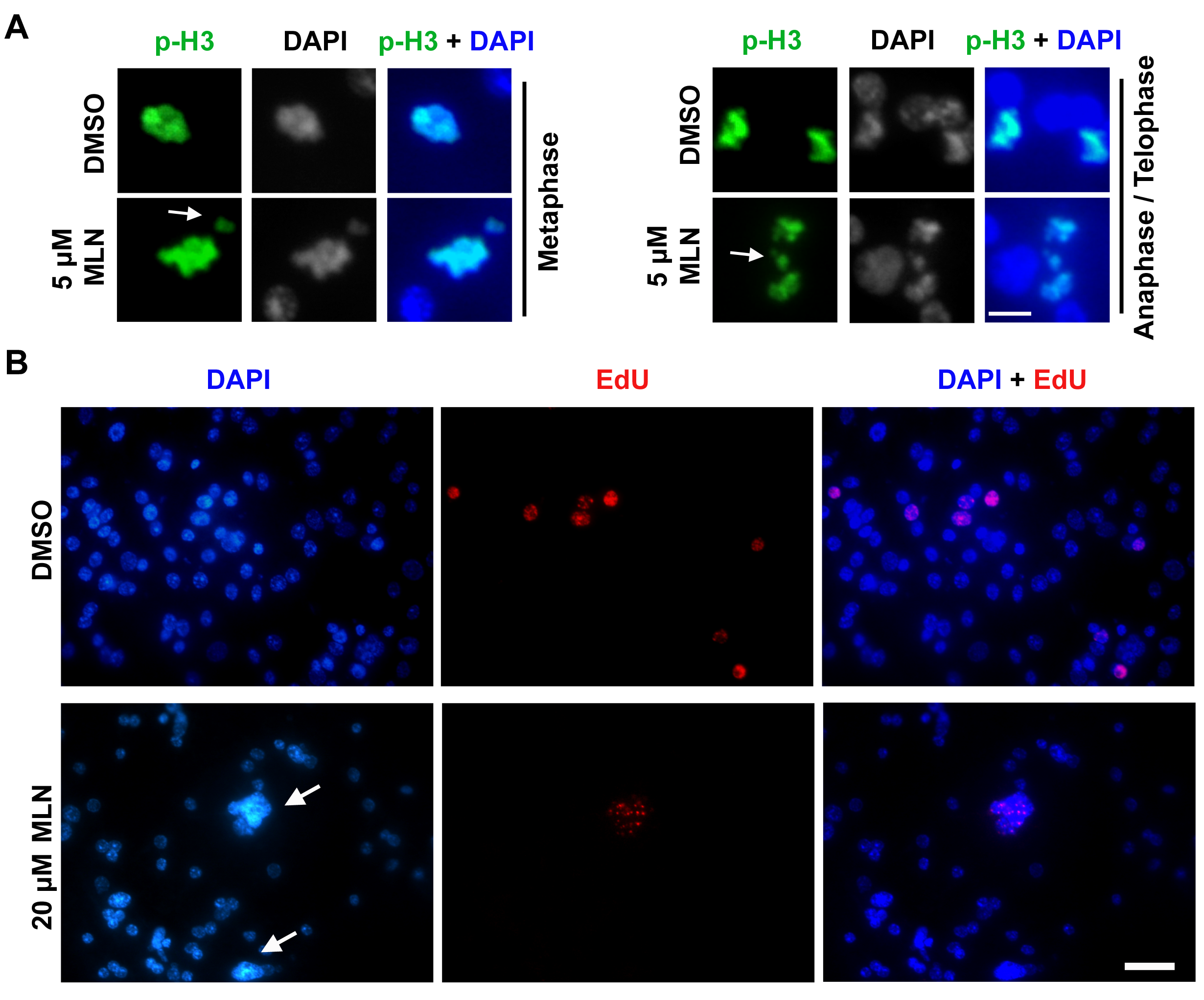

Supplement: S7 Fig — (A) Low concentration of MLN8054 causes chromosomal defects in the mitotic germinative cells. Metacestode vesicles were treated with 5 μM of MLN8054 (MLN) or DMSO for 24h. Blue: DAPI. Green: phospho-Histone H3 (Ser10). Arrows indicate the misaligned or lagging chromosomes during metaphase and anaphase / telophase. Bar = 5 μm. (B) High concentration of MLN8054 induces multinucleated germinative cells. In vitro-cultivated metacestode vesicles were treated with DMSO or 20 μM MLN8054 (MLN) for 9 days followed by 4-hour pulse of EdU (red). Arrows indicate the multinucleated cells. Bar = 25 μm. (TIF) [file pntd.0007425.s007.tif]

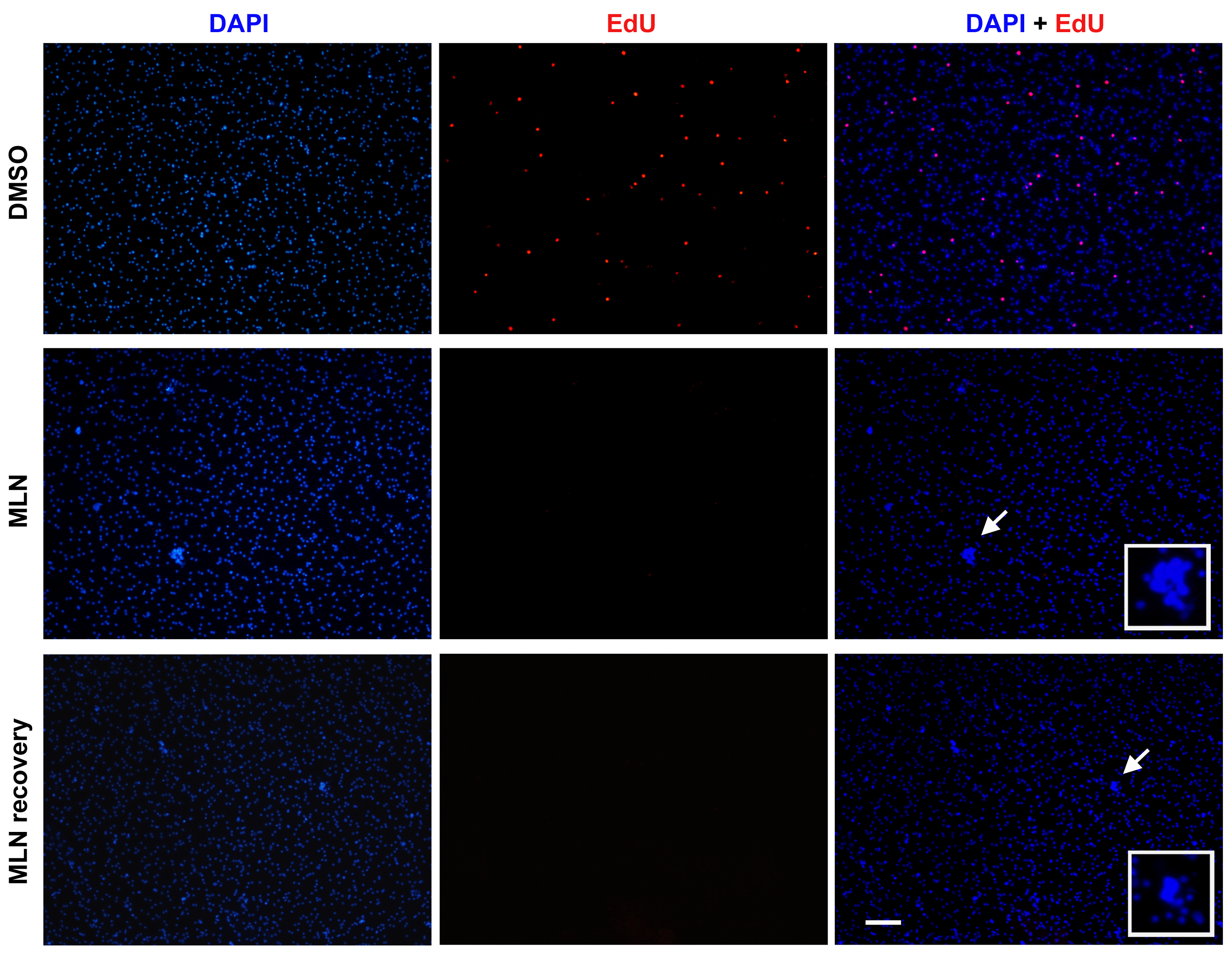

Supplement: S8 Fig — Vesicles were treated with 5 μM MLN8237 for 14 days (MLN) or allowed to recover for 3 days in drug-free media after 14 days of treatment (MLN recovery), and then administrated to 4-hour pulse of EdU (red). DAPI was used for nuclei staining (blue). Inserts are shown as the magnified view of the multinucleated cells. Bar = 100 μm. (TIF) [file pntd.0007425.s008.tif]
